# Integrative Analysis of lncRNAs in Kidney Cancer to Discover A New lncRNA (*LINC00847*) as A Therapeutic Target for Staphylococcal Enterotoxin tst Gene

**DOI:** 10.22074/cellj.2020.6996

**Published:** 2020-09-08

**Authors:** Maryam Safarpour-Dehkordi, Abbas Doosti, Mohammad-Saied Jami

**Affiliations:** 1 Department of Biology, Faculty of Basic Sciences, Shahrekord Branch, Islamic Azad University, Shahrekord, Iran; 2 Cellular and Molecular Research Center, Basic Health Sciences Institute, Shahrekord University of Medical Sciences, Shahrekord, Iran; 3 Department of Neurology, David Geffen School of Medicine, University of California Los Angeles (UCLA), USA

**Keywords:** Apoptosis, Long Non-Coding RNA, microRNA, mRNA, TSST-1 Toxin

## Abstract

**Objective:**

Bacterial toxin can cause cell death through induction of apoptosis in cancer cell lines as well as changes in the expression patterns of long non-coding RNAs (lncRNAs) and genes. In the present study, the effect of tst gene on ACHN cell lines was reported along with proposing a novel pathway of apoptosis in kidney cancer.

**Materials and Methods:**

In this experimental study, effective lncRNAs and genes were predicted from different criteria for renal cell carcinoma (RCC) by bioinformatics methods and lncRNA-miRNA-mRNA interaction was constructed; then the effect of Staphylococcus aureus tst gene on induction of apoptosis pathways on ACHN and HDF cell lines was investigated.

**Results:**

After creation of lncRNA-miRNA-mRNA interaction, changes in expression levels of lncRNA LINC00847 (P=0.0024) and PTEN gene (P=0.0027) were identified, as potential apoptosis biomarkers for kidney cancer, after treating ACHN cell line by pCDNA3.1 (+)-tst compared to the empty vector. In contrast, there was no statistically significant difference in DICER1 expression levels in ACHN-tst cell (P≥0.05). In addition, transfection by pcDNA3.1 (+)-tst could increase ACHN cell apoptosis level (P<0.0001) compared to the pcDNA3.1 (+) group; but no significant effect was observed on normal cells.

**Conclusion:**

It is suggested that lncRNA LINC00847, discovered in this study, could provide a new landscape for researches aimed to determine relationship between functional lncRNA and RCC pathways. pcDNA3.1 (+)-tst was found to increase apoptosis in the transfected cells.

## Introduction

Nowadays, cancer not only is known as one of the most common health problems, but also is prominent cause of death in societies all over the world (1). World Health Organization (WHO) reports that kidney cancer as an urologic cancer ranks first among the malignant tumors (2). Kidney cancer is the most fatal genitourinary cancer and the most significant cancer due to the new known advances on genetic mutations using the knowledge obtained from targeted systemic cures (3). Unlike the other types of disease, kidney cancer is not a single disease and according to the scientists, kidney cancer includes different types of malignancy each of which has different clinical course, responding differently to treatment, different histology and it is caused by different genes (4). Increasing prevalence of kidney cancer has been observed in different countries during the past decades, but it is difficult to treat it, because of the limited evidences for its evaluation (5, 6).

In adults, kidney cancer occurs as a result of malignant tumors increasing from renal pelvis and renal parenchyma. On the other hand, in children, kidney cancer is caused by Wilms tumor (nephroblastoma). Prevalence of nephroblastoma is about 1.1% compared to all kidney cancers. Almost all renal pelvis cancers are transitional cell carcinoma. In 90% of kidney carcinomas, adenocarcinomas arise essentially in renal parenchyma (7). There are some risk factors for kidney cancer, such as cigarette smoking, obesity, hypertension, other preexisting conditions, reproductive and hormonal factors, physical activity, diet and beverages, occupation and the environment (8). Renal cell carcinoma (RCC) is caused by the cancer originated from renal tubular epithelial cells accounting for majority of kidney cancerrelated deaths. RCC is the ninth most common types of cancer in the world accounting for ~90% of all kidney neoplasms and 2-3% of adult malignant tumors. Despite extensive research about this carcinoma, few facts have been introduced about the role of RCC-specific long noncoding RNAs (lncRNAs) (2, 9).

lncRNAs activated in cytosolic or nuclear fractions have more than 200 bp sequence length (10, 11). lncRNAs have been known as a new element, transcribed in nuclear genome using new genome sequencing techniques. Mounting evidences approved tumorigenesis role and regulation of gene expression at the various levels of lncRNAs in kidney cancer. This gene expression might appear through transcription, post-transcription processing and chromatin modification (12). New recent approaches indicated that lncRNAs illustrate a pleiotropic pattern in different human diseases. For instance, they are involved in promotion, progression and initiation of tumors (13).

Bacterial toxins have a great therapeutic potential to treat the cancer. In several studies (*in vitro* and *in vivo*), these toxins showed an effective cell-killing capacity for cancer cells. *S. aureus* is one of main human pathogens causing apoptosis during infection. Atopic dermatitis and sepsis are examples of diseases in which the *S. aureus* affects intensity and result of a disease by inducing apoptosis. Intensity of sepsis caused by *S. aureus* is related to staphylococcal toxins with properties of a super antigen such as Toxic Shock Syndrome Toxin-1 (TSST- 1) of *tst* gene. TSST-1 stimulates host immune system and causes release of interleukin (1 and 2), activating a significant amount of T-cells and tumor necrosis factoralpha (TNF-a) (14-17).

Kidney cancer is one of the most common cancers diagnosed in the world in recent decades. There are limited techniques for diagnosis and treatment of this disease. Like other types of cancer, kidney cancer is resistant to treatment methods including chemotherapy and radiation therapy, highlighting the need for identification (ID) of new biomarkers and treatment methods.

Thus, the present study was carried out to discover a new potential apoptosis pathway and integrate Staphylococcal *tst* gene in ACHN cancer cell line to measure apoptosis and expression level of the lncRNAs and related genes.

## Materials and Methods

### Recognition of the expressed lncRNAs and miRNAs

In this experimental study (The study was approved by Islamic Azad University, Shahrekord, Iran), as the first step to show contribution of lncRNA in kidney cancer, an online database was used to predict differentiallyexpressed genes. ID, transcripts and chromosomal locations of every lncRNA were recovered from Ensembl GRCh37 for more analysis. Total lncRNAs were recruited from HUGO Gene Nomenclature Committee (http://www. genennames.org). Kidney cancer dataset was recognized from TCGA (The Cancer Genome Atlas) at the cBioPortal for Cancer Genomics including 1,105 samples (http:// www.cbioportal.org/). FASTA format of each lncRNA was located into LncDisease software and the lncRNAs with higher expression profile in kidney cancer were selected. The Ensembl GRCh37, HUGO and miRWalk servers were used to select microRNAs (miRNAs). Eventually, the disease-associated lncRNAs were identified through miRNA interactions. Additionally, the Human microRNA Disease Database (HMDD) was used to conduct more studies on kidney cancer miRNAs, based on tool for microRNA set enrichment analysis (TAM) (18) method.

### Analysis of interaction between lncRNA and miRNA

Regarding analysis of binding of folded lncRNAs to folded miRNAs, bioinformatics tool of RegRNA 2.0 (http://regrna2.mbc.nctu.edu.tw/detection.html) was used to identify lncRNA-miRNA interaction. Protein sequence was provided using the NCBI database (National Center for Biotechnology Information). The least folding free energy was regulated under <-25 and system score was set at >160 to predict miRNA target sites. An increased score represents the ability for stronger binding. lncRNAs falling above the 15% alteration frequency were selected among many lncRNAs expressed in kidney cancer. Homo-sapiens lncRNA sequences were also searched in the NCBI database.

### miRNA target checking and making the lncRNAmiRNA- mRNA network

Investigative analysis of PicTar (http://pictar.mdc-berlin. de/cgi-bin/PicTarvertebrate.cgi), TargetScan (http://www. targetscan.org/vert_72/) and microcosm Targets (http://ebi. ac.uk/Enright-srv/microcosm/htdocs/targets/v5) databases were used to identify the genes for targeting through screening miRNAs. All of the genes determined using three databases were used to restrict number of false positive results. It was also confirmed that lncRNAs exhibited alterations by >15%, until maximum clarity in the network diagram. Cytoscape 3.6.0 software was used to visualize lncRNA-miRNA-mRNA interaction of significant genes (http://www.cytoscape.org/ download.php).

### Gene ontology analysis

GO enrichment of target genes was performed using the Enrichr to further study biological pathways of the genes involved in RCC.

### Recombinant plasmid preparation and confirmation

The mammalian expression vector, pcDNA3.1(+) containing *tst* encoding gene was purchased from GenRay Biotechnology (China) and the pcDNA3.1(+) (Invitrogen, USA) plasmid was used in this program as empty plasmid. The recombinant vector (pcDNA3.1(+)-*tst*) was digested using the restriction enzymes *NotI* and *EcoRV* (both from New England BioLabs, USA) to confirm the presence of *tst* gene in the recombinant plasmid.

### Cell transfection

Human renal cell adenocarcinoma (ACHN) and human dermal fibroblasts-normal (HDF) cells were provided from the National Cell Bank of Iran (Pasteur Institute, Iran). The cells were cultured in RPMI-1640 medium with 10% heat-inactivated fetal bovine serum (FBS, Gipco, USA), 100 U/ml penicillin and 100 μg/ml streptomycin (Invitrogen, USA) at 37˚C in a humidified atmosphere containing 5% carbon dioxide (CO_2_). Transfection of ACHN and HDF cells were carried out in 6-well plate according to the instructions for the Lipofectamine 2000TM reagent (Invitrogen, USA). Two micrograms of the pcDNA3.1(+)-*tst* and 2 μg of the empty pcDNA3.1(+) were transfected separately into cells. The transfected cells were selected with 600 μg/ml G418 (Invitrogen, USA) (19). In addition, there was one group from each cell, cultured in the same condition in 6-wells plate without any transfection. The cells were treated with G418, as control groups to assess the accurate performance of this aminoglycoside antibiotic.

### Annexin V-FITC assay

Cell apoptosis caused by recombinant (pcDNA3.1(+)- *tst*) and empty vector (pcDNA3.1(+)) was measured using FITC Annexin V Apoptosis Detection Kit I (BD Biosciences Pharmingen, USA) by flow-cytometer. Experiments were done in duplicate; briefly, 3×10^5^ of each cell (ACHN and HDF) were washed twice with icecold phosphate buffer solution (PBS, BIO-IDEA, Iran). Then, the cells were resuspended in 100 μl of 1X binding buffer (provided in the kit) and 100 μl of suspended cells was transferred into flow-cytometer micro-tube. They were next stained with 5 μl of FITC- Annexin-V (10 mg/ ml) and 10 μl of propidium iodide (PI, 50 mg/ml, BD Biosciences Pharmingen, USA). After incubating the cells at 25˚C for 15 minutes in the dark, 400 μl binding buffer was added and the solution was analyzed by a flow cytometer apparatus (BD, USA).

### RNA isolation and cDNA synthesis

RNX-Plus reagent (SinaClon, Iran) was used to isolate total RNA, according to the manufacturer’s protocol. RNA was quantified, and the concentration and purity were measured based on absorption rate of 260/280 nm using a Nanodrop spectrophotometer (Nanodrop 2000, Thermo Scientific, USA). Total RNA samples were treated with RNase-free DNase (Thermo Scientific, USA) before quantitative reverse transcription polymerase chain reaction (qRT-PCR). A RevertAid First Strand cDNA Synthesis Kit (Thermo Scientific, USA) was used to synthesize complementary DNA (cDNA) from total RNA. A PCR test was applied using *tst* specific primers on cDNA to confirm *tst* gene expression after lipofection. Primer sequences were as follows:

*tst*-

Sense: 5´-GCACAAACGACAACATTAAGGACC-3ˊ

Antisense: 5´-TTGTCCGCTTTGTGTTGAGGTC-3ˊ.

### Quantitative reverse transcription polymerase chain reaction analysis

Transcription levels were measured in triplicate by qRTPCR using SYBR®Premix Ex TaqTM II kit (TaKaRa, Japan). Measurements were performed using *LINC00847, PTEN *and *DICER1* specific primer pairs (in ACHN cell line) in Rotor-Gene 6000 Real-Time PCR Machine (Qiagen, Germany). *LINC00847* was evaluated as proapoptotic lncRNA in HDF cells. *GAPDH* was monitored as a reference gene and expression level of the specific genes was normalized according to *GAPDH* transcript. Different transcription levels were calculated by using 2^-ΔΔCt^ method (20). Primer sequences were as follows:

*LINC00847*-

Sense: 5´-AACGCTGCCTCTGTGGAAGTCTC-3ˊ

Antisense: 5´-CGCTCTGCTCTCCCGCCATC-3ˊ,

*PTEN*-

Sense: 5´-ACACGACGGGAAGACAAGTT-3ˊ

Antisense: 5´-CTGGTCCTGGATTGAAGAAGT-3ˊ,

*DICER1*-

Sense: 5´-GTGCGAGAATTGCTTGAA-3ˊ

Antisense: 5´-CACAGTGACTCTGACCTT-3ˊ,

*GAPDH*-

Sense: 5´-GCCAAAAGGGTCATCATCTCTCTGC-3ˊ

Antisense: 5ˊ-GGTCACGAGTCCTTCCACGATAC-3ˊ.

### Statistical analysis

All data was presented as mean ± standard error (SE). Paired Student’s t test was performed for statistical analysis. Differences with a P<0.05 were considered statistically significant. GraphPad Prism (version 8, GraphPad software, USA) was used to perform the aforementioned statistical analyses.

## Results

### Differentially expressed lncRNAs and microRNAs

**Fig.1:**
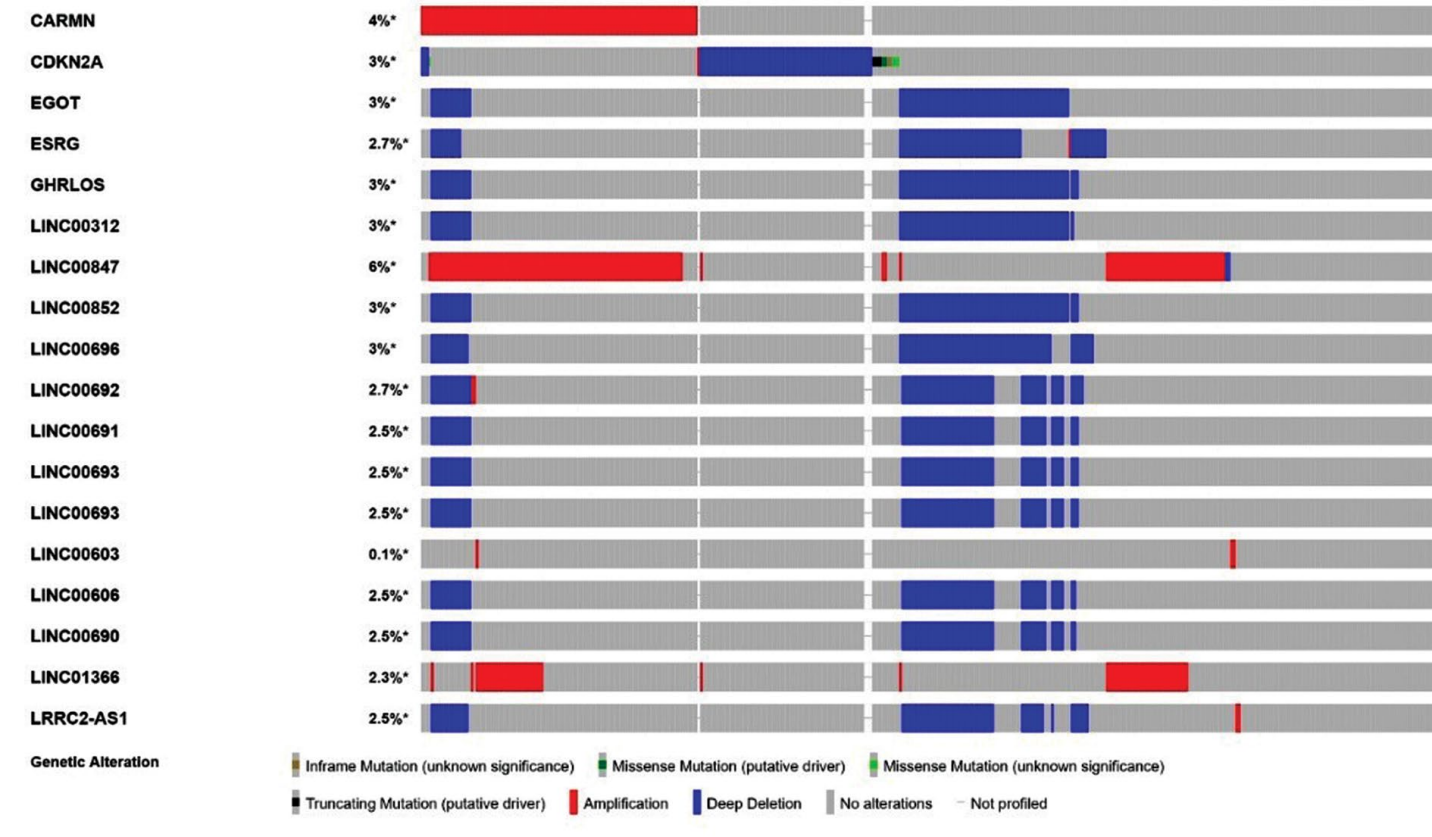
Identification of differentially expressed lncRNAs from The TCGA. Alternation frequency > 3%.

As shown in Figure 1, in this study, a total of 3994 known lncRNAs were selected as lineage-specific lncRNAs with an expressed profile above 3 (0.2%). in terms of miRNAs, 37 molecules (1.91%) were selected among 1933 miRNAs having an expressed profile above than 2.5.

### lncRNA-miRNA interactions network

**Fig.2:**
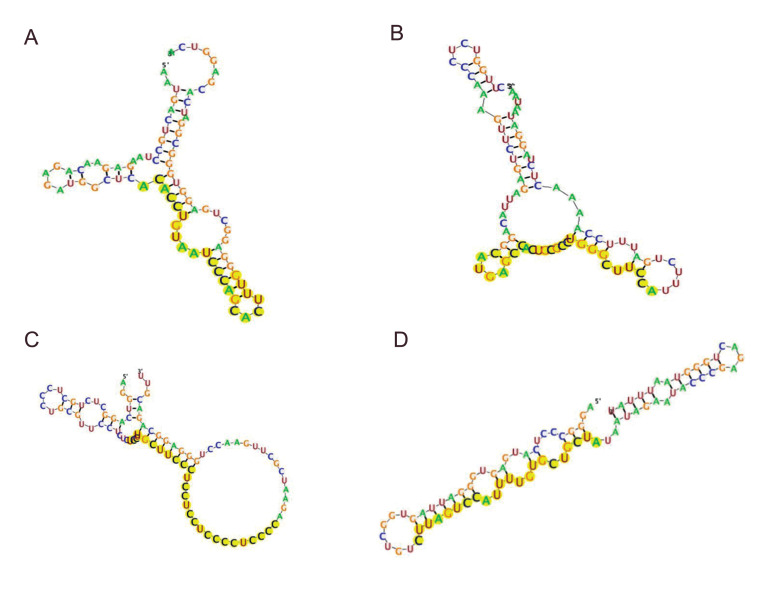
Top four RNA fold reliability data of probable lncRNA-microRNA pairs. **A.*** LINC00847-hsa-miR-93-5p.*** B.*** LINC00847-hsa-miR-671-5p.* **C. ***LINC00847- hsa-miR-4728. ***D.*** LINC00847-hsa-miR-15a-5p.* LINC, long intervening non-coding; miR, microRNA.

miRNAs have been found to regulate some of the protein-coding genes, but it is not completely known whether miRNAs can also regulate lncRNAs or not. RegRNA 2.0 database was used to analyze interaction between lncRNAs and miRNAs to identify accurate mechanism underlying the role of lncRNAs and miRNAs in kidney cancer. RegRNA 2.0 was used as a unified web server to compare mRNA sequence against insertion of homologs of regulatory RNA motifs and elements. The proposed miRNAs from this database must be intersected with kidney cancer dataset from cBioPortal. Five hindered and eighty eight miRNAs can use regulatory functions on 93 lncRNAs between differentially expressed lncRNAs with a threshold alteration frequency > 2.5%. At first, four lncRNAs with the most alteration frequencies were selected including *CARMN, LINC00847, CHRLOS, *and *LINC00852*. Results showed that only one of them interacted with RCC-related miRNA and genes. A new lncRNA named *LINC00847*, targeted by 71 miRNAs is related to kidney cancer. For example, it was predicted that *hsa-miR-15a-5p, hsa-miR-93-5p,* *hsa-miR-671-5p* and 67 other miRNAs may be used to regulate *LINC00847*. Folded RNA structure of miRNAs and lncRNAs was analyzed using RegRNA2.0 software, and Figure 2 shows limited reliability data of pair possibilities.

### Creating lncRNA-miRNA-mRNA network

**Fig.3:**
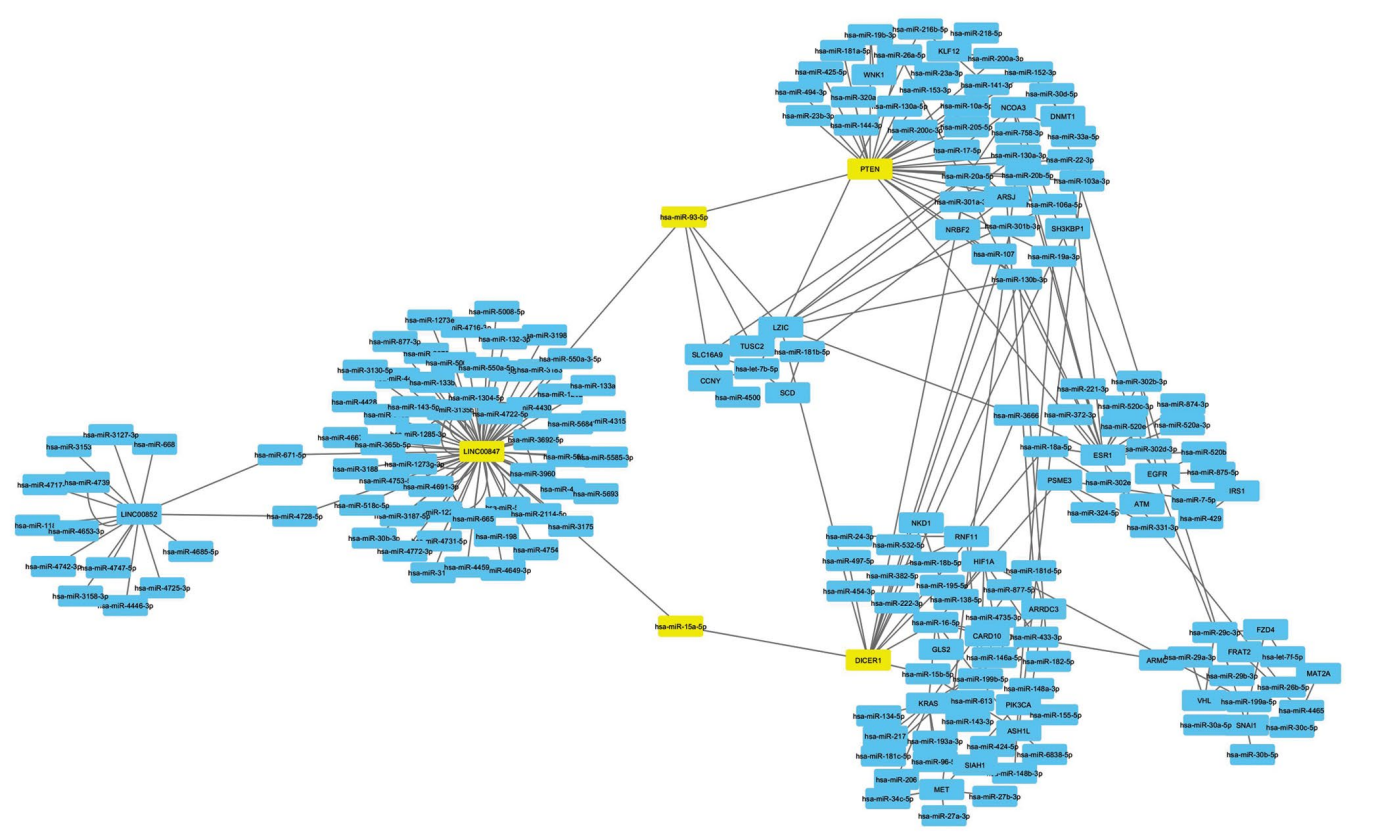
Interaction network of lncRNA-miRNA-mRNA in renal cell carcinoma (RCC). Differentially expressed mRNAs in kidney cancer were retrieved, their proximities to the selected miRNAs and lncRNAs were analyzed and visualized in Cytoscape.

In this study, 37 miRNAs were recognized to be differentially-expressed in kidney cancer. Only, miRNAmRNA pairs were simultaneously predicted by ≥ 2 applications in order to remove wrong positive rates of target prognostication. Interaction of all miRNAs with mRNAs was studied. One hundred and seventy six genes were predicted as targets of miRNA. Target genes were involved in different mechanisms of the cancer including apoptosis, cell cycle, cell proliferation and cell size. Cytoscape 3.6.0 software was used to visualize results. miRNA-mRNA network was created in this study as presented in network diagram (Fig .3), multiple miRNAs can target one gene. Regulation of *LINC00847-hsamir- 15a-5p-DICER1* was observed as a new pathway in kidney cancer according to lncRNA-miRNA-mRNA regulatory network constructed in this study.* LINC00847- hsa-miR-93-5p-PTEN* was also identified as another new pathway in kidney cancer. In the next step, the effects of *tst* gene on expression level of these identified genes were investigated.

### Functional enrichment analysis

**Fig.4:**
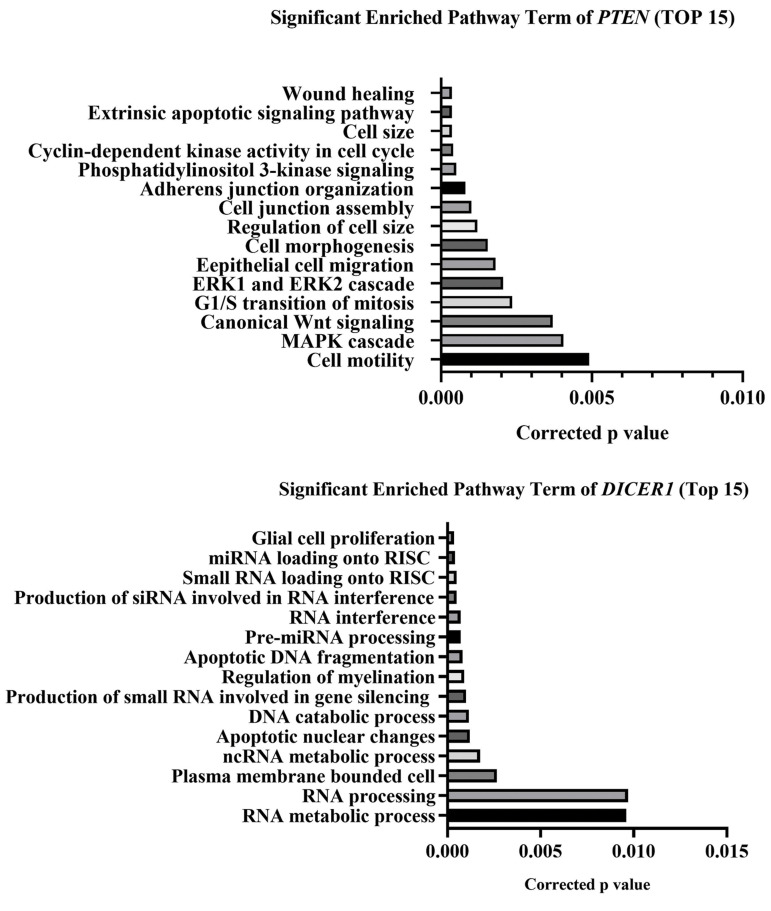
Enrichment pathway analysis. Top 15 pathways for the *PTEN* and* DICER1* genes based on biological process pathways. The lower P value is the most important activities of the genes that are shown on the top of graphs of the *PTEN* and *DICER1* genes.

Functional enrichment analyses, such as biological processes, were performed for *PTEN* and *DICER1* genes. GO class enrichments, according to threshold of enrichment, were rated with scores >1.0 and P<0.05. Enrichment genes may contribute to multiple biological processes including apoptotic signaling pathway, apoptotic DNA fragmentation, cell cycle and cell size, as shown in Figure 4.

### Confirmation of recombinant plasmid

Presence of *S. aureus* *tst* gene in the pcDNA3.1 (+)- *tst* recombinant vector was confirmed by EcoRV/ *NotI* restriction enzymes double digestion. Therefore, two fragments of 5 kb and 740 bp were observed after double digestion of pcDNA3.1(+) plasmid and *tst* gene, respectively (data not shown).

### Flow cytometry assay

**Fig.5:**
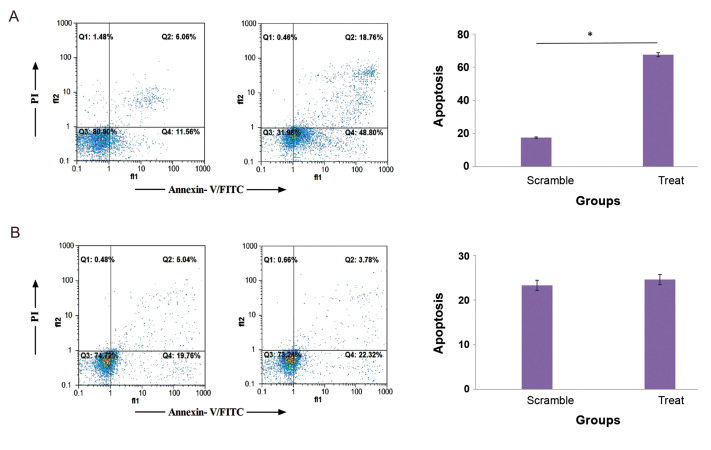
The results of apoptosis assay by FITC Annexin V for ACHN and HDF cells (Scale of axis: percentage (%)).** A.** Percentage of death in the ACHN treated group is 68.02%, after detection by flow cytometry assay, while it is 19.1% in the control group. **B.** The *tst*-treated HDF cells showed no statistically significant apoptotic cell death, compared to the control group. FITC; Fluorescein isothiocyanate, ACHN; Human renal cell adenocarcinoma, and HDF; Human Dermal Fibroblasts.

Results of flow cytometry experiments showed that apoptosis and necrosis in ACHN cells transfected with pcDNA3.1 (+)-*tst* recombinant vector were increased significantly (P<0.0001) compared to the control group (cells with empty plasmid). After *tst* treatment, death percentage of ACHN cells was clearly increased. Flow cytometry results showed that 68.02% of *tst* -treated ACHN cells were dead (due to necrosis and apoptosis), while 19.1% of cell death occurred in the control group (P<0.05, Fig .5A). In contrast, no statistically significant difference was observed in apoptosis and necrosis of HDF cells transfected with the pcDNA3.1(+)-*tst* recombinant vector (as normal cells) compared to the pcDNA3.1(+) plasmid (P=0.3246, Fig .5B).

### Mammalian expression of *tst* gene

ACHN and HDF cells transfected with recombinant pcDNA3.1 (+)-*tst* expression vector were harvested 10 days post-transfection. RT-PCR results showed that 207 bp fragment was amplified for *tst* gene, suggesting that recombinant plasmid was successfully transfected into ACHN and HDF cells (data not shown).

### Significant changes in the expression level of specific lncRNA and related genes in RCC cell line

**Fig.6:**
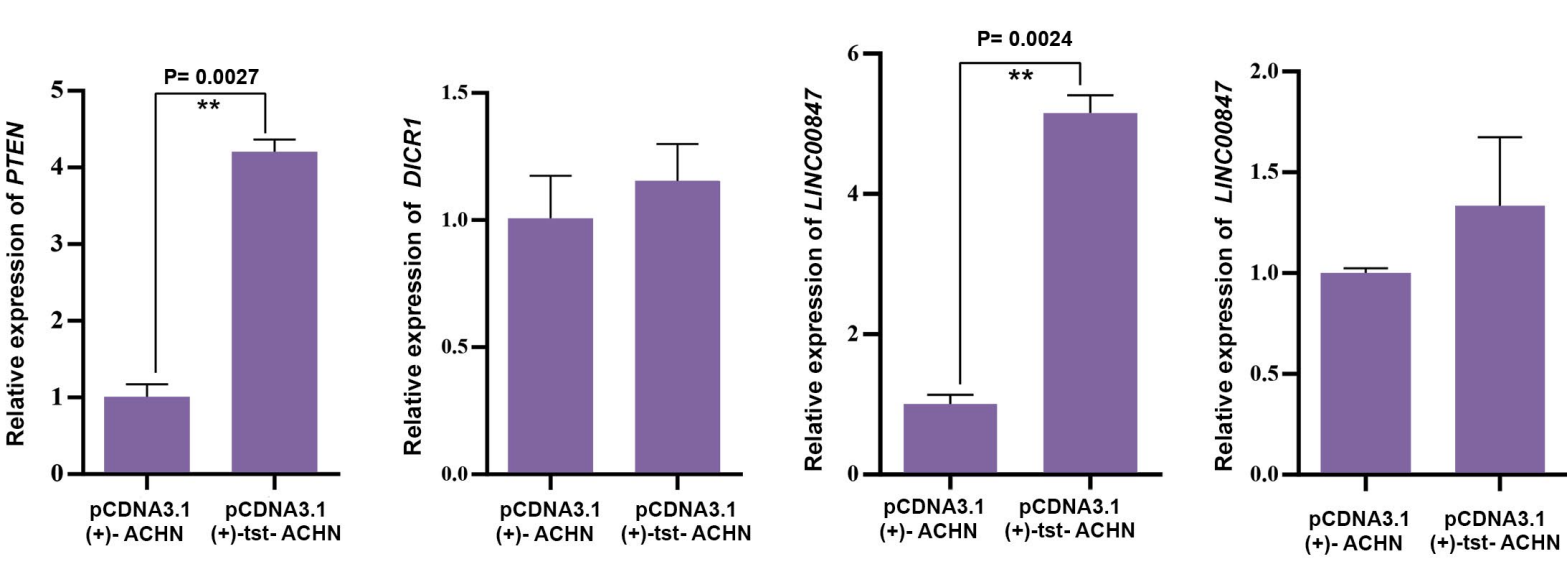
Expression levels of *PTEN, DICER1,* and *LINC00847* in the ACHN kidney cancer cell and *LINC00847* in the HDF normal cells treated by *tst* gene. Relative expression of the genes were examined by quantitative reverse transcription polymerase chain reaction (qRT-PCR) and they were compared to the treated and control cells by ΔΔCt method. Asterisks show significant differences to the controls (*; P<0.05, **; P<0.01, ***; P<0.001). ACHN; Human renal cell adenocarcinoma, and HDF; Human Dermal Fibroblasts. ACHN; Human renal cell adenocarcinoma, and HDF; Human Dermal Fibroblasts.

Expression levels of the selected lncRNA and related genes in RCC cell line were measured after lipofection, compared to the empty plasmid group. qRT-PCR results showed a significant increase in the *LINC00847* expression in ACHN-*tst* group compared to pcDNA3.1(+) group (P=0.0024). A significant difference was found in the expression intensity (>3-fold change); therefore, *LINC00847* was introduced as a pro-apoptotic gene. Moreover, *PTEN* gene related to apoptosis pathway and their exclusive miRNA was increased in the ACHN-*tst* group compared to the pcDNA3.1 (+) group (P=0.0027 and ≥3-fold change). In contrast, no statistically significant difference was found in the expression levels of *DICER1* in the pcDNA3.1 (+)- *tst* transfected cells compared to the pcDNA3.1(+) group (P=0.4498, Fig .6). Additionally, no statistically significant difference was observed in the expression levels of *LINC00847* in the pcDNA3.1(+)-*tst* -HDF cells compared to the pcDNA3.1(+) group (P=0.3043, Fig .6). This indicates that *tst* gene had no statistically significant effect on the normal cells.

## Discussion

In this study, the effects of non-coding RNAs were analyzed to prepare a network for elucidating lncRNAmiRNA- mRNA interaction in kidney cancer followed by measuring efficiency of recombinant plasmid in apoptosis of cancer cell lines. General analysis procedures have been used to find unique expressed genes, lncRNAs and miRNAs in biological processes or diseases. Few studies have been conducted on interactions within lncRNAs, miRNAs and target genes in kidney cancer. In this study, the obtained results regarding the expression level used to recognize abnormally expressed miRNAs and lncRNAs, in addition to the interaction network of lncRNA-miRNAmRNA, was provided in kidney cancer. Data of miRNA and lncRNA expression levels related to kidney cancer were achieved by the Cancer Genome Atlas, to find genes which are likely to be related to the cancer. It is suggested to explain more completely the process of lncRNAs in RCC, in future empirical researches. Initially, it was believed that lncRNAs are ‘transcription noise’. However, lncRNAs have now been identified as significant players in gene regulation and they are related to a majority types of cancer (22, 23).

RegRNA software was used to predict interactions between lncRNAs and miRNAs. A total of 176 genes were targeted by miRNAs in this study, according to the results of ≥ 2 different algorithms, *hsa-miR-93-5p* and* hsa-mir- 15a-5p* are the main elements in the constructed network. Created network showed an interaction within lncRNAs, miRNAs and mRNAs in relation to the development or occurrence of RCC. Our results (a relationship was identified between lncRNAs and mRNAs) showed that lncRNAs were related to miRNAs and vice versa. It was hypothesized that lncRNAs may also be related to clinical and pathological features of kidney cancer, just like miRNAs. Results of this study showed that one lncRNA, termed *LINC00847*, exhibited the possibility of interaction with *PTEN* and *DICER1* genes. Enrichr was used to analyze GO biological process in order to further study biological effects of aberrantly-expressed *PTEN* and *DICER1 *in kidney cancer. Prognostication data showed that these genes may be involved in some biological processes including apoptotic signaling pathway, apoptotic DNA fragmentation, cell cycle and cell size. Nowadays, functional roles of most of the lncRNAs are obscure in cancers, but the role of *HOTAIR* is well-known. Liu et al. (24) showed that *HOTAIR* acting as a competing endogenous RNA (CeRNA) was a target of *miR-331-3P*. It can impose an additional level of posttranscriptional regulation and thereby modulating derepression of *HER2*. Therefore, lncRNAs, miRNAs and mRNAs showed a regulatory network to co-interact with gene expression.

Hence, more disease-associated lncRNAs can be found using these methods in particular cells. The present report provided a new perspective into molecular pathway of RCC. However, these mechanisms have some limitations. For example, all of the miRNAs cannot be concurrently registered in prediction software algorithms. On the other hand, in vivo and in vitro studies will be required later due to lncRNA-miRNA-mRNA regulatory network proposed in this report, using a bioinformatics approach. Herein, *LINC00847, PTEN* and *DICER1* were identified based on the different servers. In fact, it was found that *LINC00847, PTEN* and *DICER1* are involved in apoptotic pathways in kidney cancer, and interaction network of lncRNAmiRNA- mRNA in RCC was also created. Correlation of these genes is based on the significance level of P value. On the other hand, this study was conducted to investigate the effect of *tst* gene on apoptosis. For this reason, expressions of *LINC00847, PTEN *and *DICER1* were investigated after lipofection. Therefore, a new therapeutic method was designed. Bacterial toxin can cause cell death by inducing apoptosis in cancer cell lines (25). As a result, efficiency of recombinant plasmid was studied in cancer cell lines. The pcDNA3.1 (+) mammalian expression vector was used to insert encoding *tst* gene. ACHN and HDF cell lines were transfected with pcDNA3.1 (+)-*tst* and pcDNA3.1 (+) as empty plasmid, and cell death was evaluated in the tested cells using Annexin V/PI staining and flow cytometer to measure the extent of necrosis and apoptosis. Our results significantly showed more cell death in the ACHN cell lipofected with pcDNA3.1 (+)- *tst* compared to the control groups. Cell death percentage caused by apoptosis in treated and control ACHN cells was equal to 68.02% and 19.1%, respectively. While, there was no significant necrosis and apoptosis in the HDF cells (as normal cells) in comparison with the control groups.

qRT-PCR of *LINC00847* showed up-regulation in ACHN treated cells compared to the controls. To find out whether lncRNA related to apoptosis (*LINC00847*) can influence on expression of the related genes or not, *PTEN* and *DICER1 *gene expressions in the treated ACHN cells were studied, in comparison with the controls. Results obtained from *PTEN* expression indicated that this gene is indeed responsible for the increased level of apoptosis. So that, *PTEN* expression levels in the ACHN cells transfected with pcDNA3.1(+)-*tst* was significantly increased in comparison with the ACHN cells transfected with empty pcDNA3.1(+) vector. *PTEN* is known as a tumor suppressor gene, which activates apoptosis pathway by reducing intracellular phosphatidylinositol- 3,4,5-triphosphate (PIP3). Protein abundance of *PTEN* decreases PIP3 level in cells and causes a decrease in the Akt protein on plasma membrane. This in turn decreases cell proliferation potential and increases apoptosis (26). Results of this study showed that expression level of *PTEN* was increased in ACHN-*tst*, compared to pcDNA3.1 (+) group, indicating that apoptosis induction was achieved in ACHN-*tst* group. Therefore, it can be stated that an increase in the expression of these genes could cause cell death in the *tst*-treated ACHN cell line. In contrast, expression levels of *DICER1* gene showed no statistically significant difference between *tst*-treated ACHN cells and pcDNA3.1 (+) group. Insufficient capabilities of the software to suggest probable lncRNA-miRNA-mRNA networks might be a reason for high false positive/negative outputs and finding no significant difference in the expression levels of *DICER1*. Expression of *LINC00847*, as a pro-apoptotic lncRNA, in the *tst*-treated HDF cell showed no statistically significant difference between the *tst*-treated cells and pcDNA3.1 (+) group. This indicates that *tst* gene had no effect on the normal cells.

As previously mentioned, bacterial toxins have great therapeutic potential to treat the cancers. Bacterial toxins are the most obvious cytotoxic agents, because these genes are native to bacterial physiology. Bacterial toxins are used to induce apoptosis during infection and they are presently considered to be important in disease processes (16, 27). Yu et al. (28) studied the effects of *S. aureus* toxins, SEB and α-toxin, on ECV304 cells. Results of this study showed apoptosis induction and increase in TNF-α expression, as well as activation of caspase 3 and 8 in ECV304 cells. Findings expressed that SEB and α-toxin induce apoptosis through extrinsic apoptosis pathway. In this study, considering apoptosis induction, similar results were achieved for *S. aureus* toxin TSST-1.

## Conclusion

In the current study, a variety of bioinformatic approaches were used, as a result of which a new lncRNA was discovered in the kidney cancer along with apoptosis pathways. lncRNAs and miRNAs were also found to exert regulatory effects on kidney cancer apoptosis, by influencing signaling pathways and biological process. Moreover, the effect of *tst* expression on ACHN cell line was investigated and the results were obtained regarding apoptosis induction. Expression of *LINC00847*, as a cell apoptosis-inducing lncRNA, and *PTEN* gene was upregulated in the *tst*-treated ACHN cells. These results expressed that *S. aureus* toxin TSST-1 arrested cell cycle and resulted in activation of apoptosis through regulatory lncRNA and associated genes. Generally, *S. aureus* toxin TSST-1 can be used as a therapeutic bacterial toxin to treat the cancer in future.
